# Identification and characterization of circular RNAs involved in the fertility stability of cotton CMS-D2 restorer line under heat stress

**DOI:** 10.1186/s12870-023-04706-w

**Published:** 2024-01-05

**Authors:** Ruijie Wang, Meng Zhang, Hui Wang, Liangliang Chen, Xuexian Zhang, Liping Guo, Tingxiang Qi, Huini Tang, Kashif Shahzad, Hailin Wang, Xiuqin Qiao, Jianyong Wu, Chaozhu Xing

**Affiliations:** 1 National Key Laboratory of Cotton Bio-breeding and Integrated Utilization, Key Laboratory for Cotton Genetic Improvement, Ministry of Agriculture and Rural Affairs, Institute of Cotton Research of Chinese Academy of Agricultural Sciences, 38 Huanghe Dadao, Anyang, 455000 Henan China; 2 Xiangyang Vocational and Technical College, Xiangyang, 441050 Hubei China

**Keywords:** CMS-D2 cotton, Fertility restoration, CircRNAs, Heat stress, CircRNA-mediated ceRNA network

## Abstract

**Background:**

As a vital type of noncoding RNAs, circular RNAs (circRNAs) play important roles in plant growth and development and stress response. However, little is known about the biological roles of circRNAs in regulating the stability of male fertility restoration for cytoplasmic male sterility (CMS) conditioned by *Gossypium harknessii* cytoplasm (CMS-D2) cotton under high-temperature (HT) stress.

**Results:**

In this study, RNA-sequencing and bioinformatics analysis were performed on pollen grains of isonuclear alloplasmic near-isogenic restorer lines NH [N(*Rf*_*1*_*rf*_*1*_)] and SH [S(*Rf*_*1*_*rf*_*1*_)] with obvious differences in fertility stability under HT stress at two environments. A total of 967 circRNAs were identified, with 250 differentially expressed under HT stress. We confirmed the back-splicing sites of eight selected circRNAs using divergent primers and Sanger sequencing. Tissue-specific expression patterns of five differentially expressed circRNAs (DECs) were also verified by RT-PCR and qRT-PCR. Functional enrichment and metabolic pathway analysis revealed that the parental genes of DECs were significantly enriched in fertility-related biological processes such as pollen tube guidance and cell wall organization, as well as the Pentose and glucuronate interconversions, Steroid biosynthesis, and N-Glycan biosynthesis pathways. Moreover, we also constructed a putative circRNA-mediated competing endogenous RNA (ceRNA) network consisting of 21 DECs, eight predicted circRNA-binding miRNAs, and their corresponding 22 mRNA targets, especially the two ceRNA modules circRNA346-miR159a-*MYB33* and circRNA484-miR319e-*MYB33*, which might play important biological roles in regulating pollen fertility stability of cotton CMS-D2 restorer line under HT stress.

**Conclusions:**

Through systematic analysis of the abundance, characteristics and expression patterns of circRNAs, as well as the potential functions of their parent genes, our findings suggested that circRNAs and their mediated ceRNA networks acted vital biological roles in cotton pollen development, and might be also essential regulators for fertility stability of CMS-D2 restorer line under heat stress. This study will open a new door for further unlocking complex regulatory mechanisms underpinning the fertility restoration stability for CMS-D2 in cotton.

**Supplementary Information:**

The online version contains supplementary material available at 10.1186/s12870-023-04706-w.

## Background

Circular RNAs (circRNAs) are a diverse class of endogenous noncoding RNAs, that ubiquitously exists in all eukaryotes and prokaryotic archaea [1]. CircRNAs do not have a 5′ cap structure and a 3′ poly(A) tail, form a single-stranded ring structure with covalent bonds [2], and are predominantly yielded from linear precursor messenger RNAs (pre-mRNAs) that undergo a non-canonical splicing event termed back-splicing [3]. They have diverse genome positions in pre-mRNAs and are usually divided into three classes exonic circRNAs, intronic circRNAs, and intergenic circRNAs [4]. CircRNAs were first reported in viroids of certain higher plants in 1976 [5]. Due to technical limitations of sequencing and bioinformatics, circRNAs were ignored for decades, and considered as splicing errors, transcriptional noise or reverse transcription (RT)-PCR artifacts by-product [1]. Until recently, with the advance of high-throughput sequencing technology and high-efficiency bioinformatics methods [6, 7], circRNAs have been extensively identified in all domains of life, including eukaryotic animals [1] and plants [8], prokaryotic archaea [9] and bacteria [10], and even virus [11]. Recent research has shown that circRNAs are more stable than linear RNA molecules in cells, have a longer half-life, and can resist RNase R degradation [12]. Additionally, circRNAs have also been found to present complex tissue, cell-type, or developmental-stage specific expression profiles, and their sequence features and expression patterns may be often conserved in various species [2, 13–16].

Recently, a large number of studies have discovered the important functions of circRNAs in animal and human systems. In 2013, it was reported for the first time that circRNAs can act as 'miRNA sponges' to adsorb miRNAs and further regulate downstream target genes [1, 17]. Furthermore, circRNAs can regulate gene expression [18], interact with RNA polymerase II or other RNA-binding proteins (RBPs) to function as protein sponges [19], and some can also be translated into proteins or polypeptides through translation initiation element internal ribosome entry site (IRES) or N6-methyladenosine (m^6^A) [20]. Growing evidence has revealed that circRNAs regulate various cancer development, resulting in a boom in animal circRNA research. Nevertheless, studies of circRNAs in plants lag compared with animal models, andthe regulatory mechanisms of biogenesis and molecular functions of plant circRNAs are particularly important but remain largely elusive. In plants, identification of circRNAs was first performed in the model plant *Arabidopsis thaliana* in 2014 [16]. Later, circRNAs have been gradually identified in rice [8, 21], wheat [22], maize [23–25], barley [26], cotton [27, 28], kiwifruit [29], soybean [30, 31], tomato [32–34], sea buckthorn [35] and other plant species, indicating that circRNAs are also widely present in plants. Many studies have suggested that plant circRNAs can modulate vegetative or reproductive growth and development [30, 36, 37] and the response of plants to biotic and abiotic stresses [29, 33, 38]. However, most of the large-scale sequencing research on plant circRNAs is still at the stage of circRNAs identification. So far, only several circRNAs have been elucidated with clear molecular functions [20]. For example, the circRNA from *SEPALLATA3* (*SEP3*) gene increased the abundance of exon-skipped alternative splicing variants via forming an R-loop structure with the cognate DNA and finally regulated the stamen number and petal number of *Arabidopsis* [39]. Thereafter, a lariat-derived circRNA (laciRNA) from *At5g37720* was validated by overexpressing constructs in *Arabidopsis* to be essential for plant development through regulating global gene expression [40]. These findings indicate that plant circRNAs indeed play critical roles in transcriptional and posttranscriptional regulatory processes, but in-depth validation experiments are still necessary to further illuminate the potential regulatory functions of circRNAs in plants.

Cotton (*Gossypium hirsutum* L.) is one of the most important crash crops and is widely grown worldwide [41]. Utilization of heterosis can not only effectively increase cotton yield, but also improve fiber quality and enhance stress resistance [42]. As an economic pollination control system, the utilization of cytoplasmic male sterility (CMS) for hybrid seed production can not only retrench the tedious steps of manual emasculation but also can efficiently improve the purity of hybrids [43]. In China, CMS-D2 is the main source of sterile cytoplasm for cotton "three-line" hybrids presently grown in production [44, 45]. However, the anther and/or pollen development of CMS-D2 restorer lines and hybrids are susceptible to uninterrupted high-temperature (HT) stress in summer [46], which gravely hinders the large-scale application of "three-line" hybrids, especially in China and India. Therefore, further in-depth discovering the regulatory mechanisms of pollen fertility restoration in response to HT can hold great significance in promoting "three-line" hybrid breeding in cotton. Recent studies have implicated circRNAs in the regulation of pollen development [30, 36, 37] or response to HT [38]. For instance, a total of 186 differentially expressed circRNAs (DECs) were identified at three different pollen developmental stages during the fertility transition of the photo-thermosensitive genic male sterile line in rice [36]. In soybean, a total of 2,867 circRNAs and 1,009 DECs were obtained between CMS line NJCMS1A and its maintainer NJCMS1B, and several key circRNAs have been found to play an important role in regulating CMS occurrence through signal transduction and programmed cell death pathways [30]. Similar research in *Brassica campestris* revealed 31 DECs between the Polima CMS and fertile lines involved in anther development [37]. To date, a large number of circRNAs have been identified in cotton that may be involved in the regulation of polyploidy formation [27], Verticillium wilt resistance [47], fiber development [28], and the formation of heterosis [48]. However, the relationship between circRNAs and pollen fertility of CMS-D2 restorer lines under heat stress is not thoroughly investigated.

Here, whole genome-wide identification and characterization of circRNAs using high-throughput sequencing technology were conducted to investigate the expression profiles of circRNAs in pollen grains of isonuclear alloplasmic near-isogenic restorer lines NH and SH at two environments and further explore their potential roles with pollen fertility stability under HT stress for the first time. A total of 967 circRNAs and 250 DECs were identified under heat stress. Functional annotation of the parental genes of DECs indicated that circRNAs might play crucial roles in the fertility restoration for CMS-D2 cotton through the sugar, plant hormone, and reactive oxygen species (ROS) signals. Finally, a putative circRNA-mediated ceRNA network interlinked with pollen fertility was constructed. Our results provide new insights into how circRNAs and their mediated ceRNA networks precisely regulate the fertility restoration stability for CMS-D2 cotton under heat stress, and the obtained epigenetic resources here would be valuable for further accelerating the breeding of heat-tolerant cotton restorer lines or "three-line" hybrids by improving the fertility stability through epigenetic engineering techniques to address the potential threat of current and future global warming to crop production.

## Results

### Systematic identification and characterization of circRNAs in cotton restorer lines under HT stress

Our previous studies have confirmed that the male fertility of cotton restorer line with sterile CMS-D2 cytoplasm is susceptible to continuous HT stress [46, 49, 50]. To further investigate the functional roles of circRNAs in male pollen fertility restoration process under HT stress, 12 rRNA-depleted RNA libraries from mature pollen grains of isonuclear alloplasmic near-isogenic cotton restorer lines NH (HT-tolerant) and SH (HT-sensitive) under HT at two environments were sequenced using an Illumina Novaseq™ 6000 platform. In total, approximately 1,031,802,156 raw reads (154.76 Gb) were produced, with an average of 12.90 Gb per sample (Additional file 1: Table S1). Specifically, a total of 257.29, 265.17, 270.61, and 238.72 million raw reads were generated from AP_NH, AP_SH, JP_NH, and JP_SH samples, respectively. After removing the low-quality reads, a total of 148.46 Gb clean data (valid data) were obtained and the total amount of sequences for each RNA library was more than 10 Gb with an average Q20 > 99%, and Q30 > 97% (Additional file 1: Table S1), which indicates the accuracy and depth of high-quality sequencing reads for subsequent bioinformatics analysis.

**Fig. 1:**
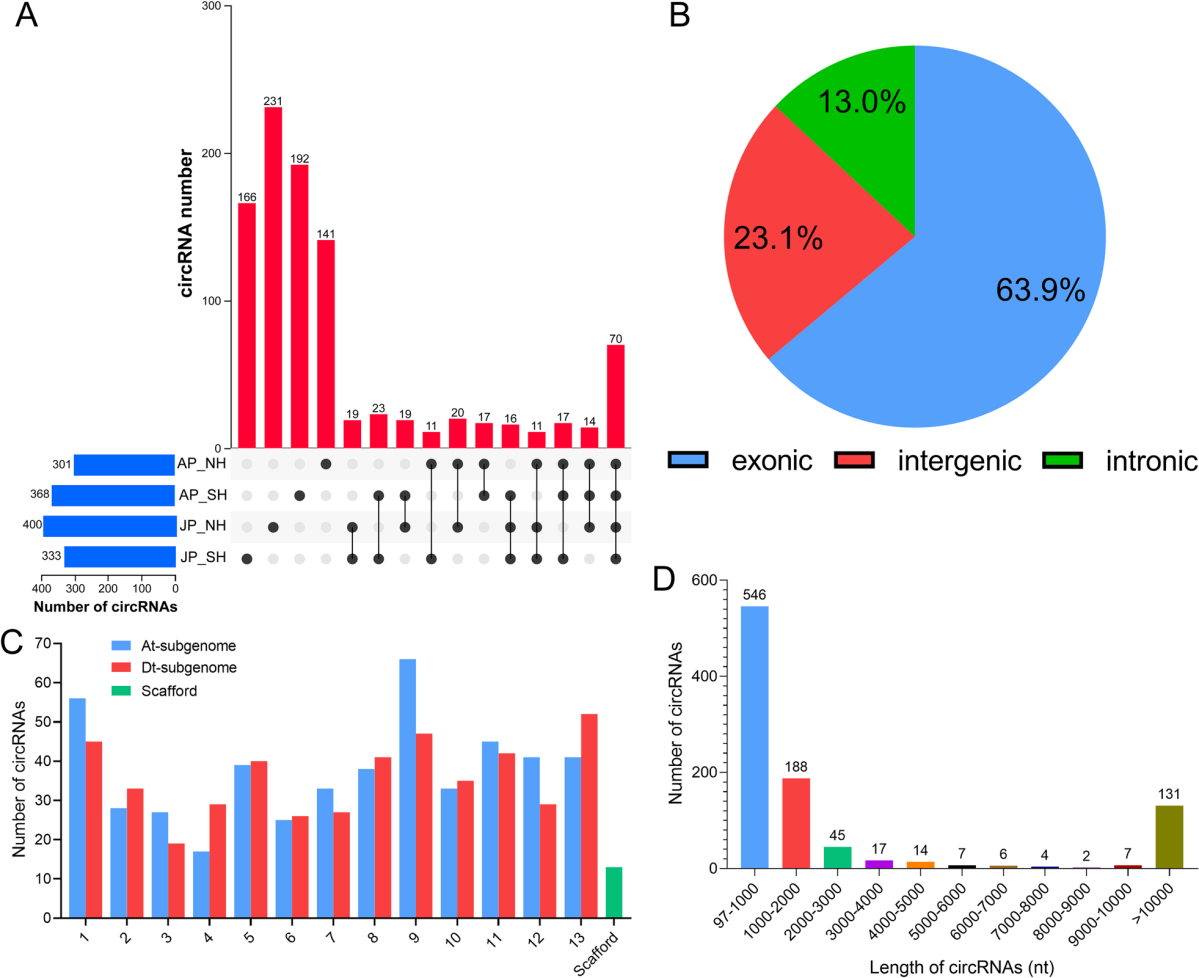
Characterization of identified circRNAs in pollen grains of two cotton restorer lines under HT stress. **A** UpSet Venn diagram showing the number of shared and specific circRNAs identified in different samples. AP_NH, NH under mild HT stress; AP_SH, SH under mild HT stress; JP_NH, NH under extreme HT stress; JP_SH, SH under extreme HT stress. **B** Source statistics of the identified circRNAs. **C** Histogram showing the number of circRNAs identified on each chromosome of upland cotton. **D** Sequence length distribution of the identified circRNAs

The application of further bioinformatic analysis yielded a total of 967 circRNAs from all 12 cotton pollen samples in the study, which included 301, 368, 400, and 333 from AP_NH, AP_SH, JP_NH, and JP_SH, respectively. Among them, 70 shared circRNAs were found in all above four samples, whereas 141, 192, 231 and 166 specific circRNAs were only identified in AP_NH, AP_SH, JP_NH and JP_SH, respectively (Fig. 1A). In all of the identified circRNAs, 618 (63.9%) were generated from exons of a single protein-coding gene (exonic circRNAs), 126 (13.0%) from introns (intronic circRNAs, ciRNA), and 223 (23.1%) from intergenic regions (intergenic circRNAs) (Fig. 1B). Specifically, the number of circRNAs on the At-subgenome chromosomes (A01-A13) of *G. hirsutum* ranged from 17 to 66, while the number on the Dt-subgenome chromosomes (D01-D13) ranged from 19 to 52. Nevertheless, the distribution of the circRNAs was even between the At- and Dt-subgenomes of *G. hirsutum* (489 and 465 circRNAs, respectively), with an additional 13 circRNAs on Scaffold in cotton (Fig. 1C). The average length of the circRNAs was 6,877 bp in cotton pollen grains, while the maximum length was 192,774 bp and the minimum length was only 97 bp. Furthermore, approximately 75.9% identified circRNAs in cotton pollen grains had length shorter than 2000 bp (Fig. 1D).

### Expression changes of circRNAs in pollen grains in response to HT stress

**Fig. 2:**
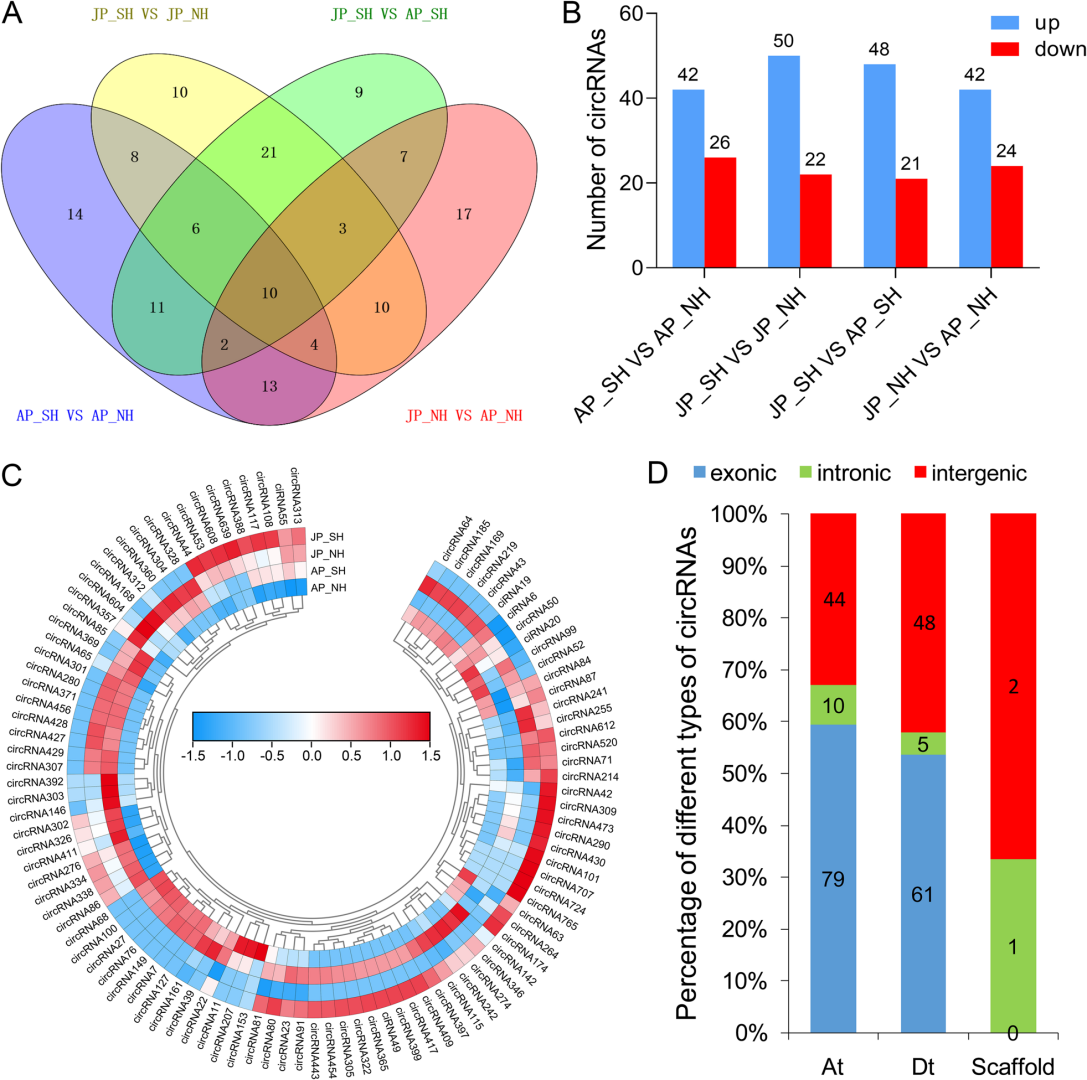
Identification of differentially expressed circRNAs (DECs) in NH and SH under mild and extreme HT stress. **A** Venn diagram showing the number of unique and shared DECs in different comparison groups. **B** Number of DECs that were up- or down-regulated in different comparison groups. **C** A bending heat map showing the relative expression levels of the top 100 DECs displayed with log_10_ (norm + 1) value. AP_NH, NH under mild HT stress; AP_SH, SH under mild HT stress; JP_NH, NH under extreme HT stress; JP_SH, SH under extreme HT stress. **D** The distribution of different types of DECs on the At- and Dt-subgenomes and Scaffolds

To determine the expression dynamics of circRNAs under HT stress, four comparisons were performed, and a total of 250 differentially expressed circRNAs (DECs) were identified, the 68 of which were from AP_SH versus (VS) AP_NH, 72 from JP_SH VS JP_NH, 69 from JP_SH VS AP_SH and 66 from JP_NH VS AP_NH. Moreover, the Venn diagram displayed that the combinations AP_SH VS AP_NH and JP_SH VS JP_NH had 28 DECs in common. This indicates that most DECs between NH and SH had differential regulation in response to HT (Fig. 2A). Therefore, DECs showed different numbers of up and down-regulated circRNAs in different comparison groups. Specifically, 42 and 24 circRNAs were up- and down-regulated, respectively, in JP_NH VS AP_NH, whereas 48 up and 21 down-regulated circRNAs were identified in JP_SH VS AP_SH. Under mild HT stress, 42 up and 26 down-regulated circRNAs were identified for the comparison of AP_SH VS AP_NH. Similarly, 50 circRNAs were up-regulated and 22 were down-regulated between NH and SH under extreme HT stress (Fig. 2B). Hierarchical cluster analysis of the top 100 DECs with TBtools software [51] revealed that expression levels of several circRNAs had obvious differences (Fig. 2C). In particular, most circRNAs between NH and SH were found to exhibit a wide range of expression changes in response to HT stress. The distribution of different types of DECs on different chromosomes determined that proportions of exonic DECs on the At- or Dt-subgenomes were relatively higher than intronic or intergenic DECs (Fig. 2D).

**Fig. 3:**
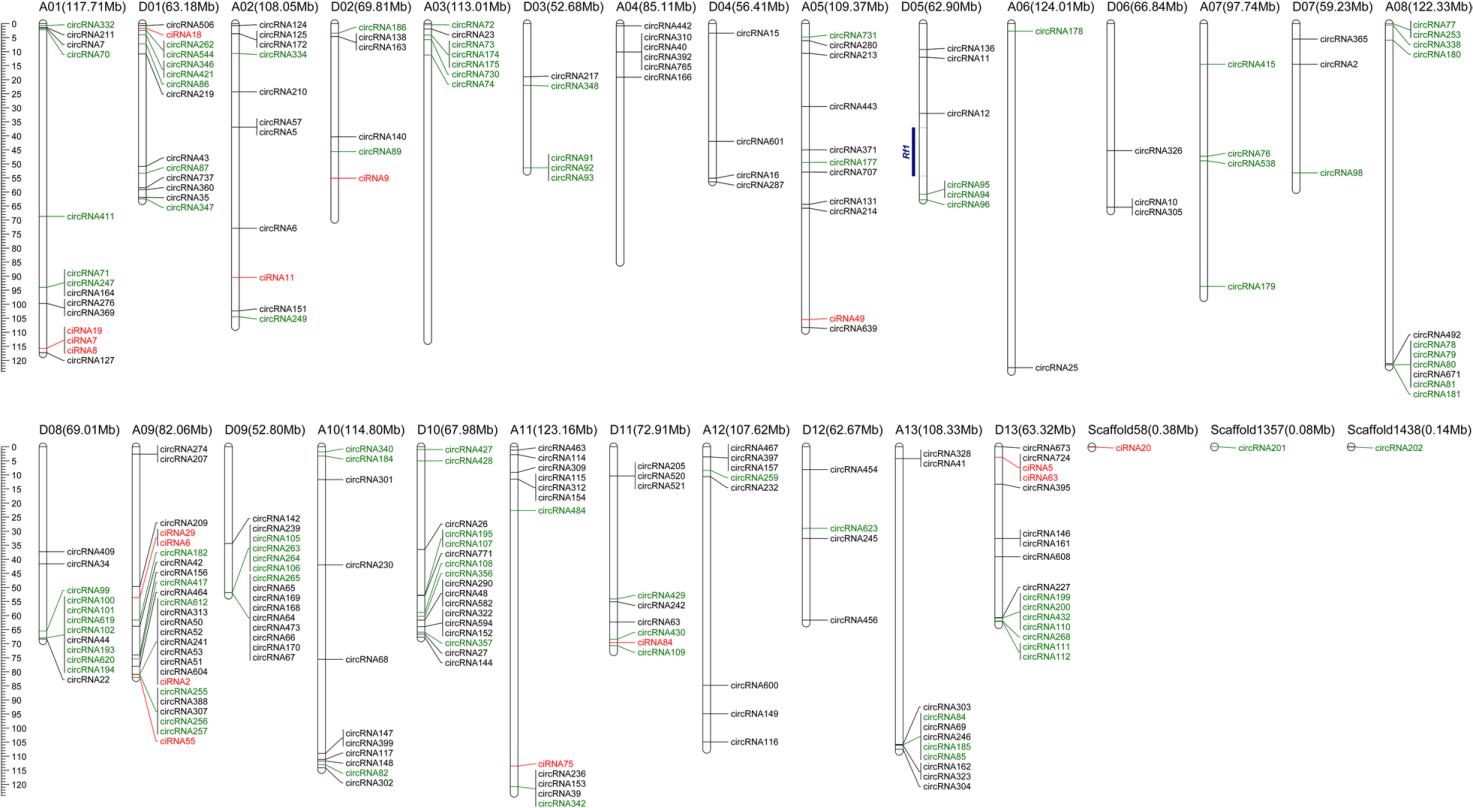
Location distribution of the identified 250 DECs on different chromosomes of upland cotton. Different colors indicate different categories of circRNAs, namely green, red, and black represent the exonic, intronic, and intergenic DECs, respectively. The blue column on the left side of the Chir_D05 chromosome signifies the mapped interval of the fertility restorer gene *Rf*_*1*_ in our previous study [52]

Besides, we further co-localized 250 DECs on the genome of upland cotton (Fig. 3). The densities of DECs in different chromosomal regions presented a difference, and most cotton pollen grains DECs in were clustered at the end of chromosomes. However, no corresponding DECs were located in the fine-mapped interval of the *Rf*_*1*_ gene [52].

### Experimental validation of circRNAs candidates in cotton pollen grains

**Fig. 4:**
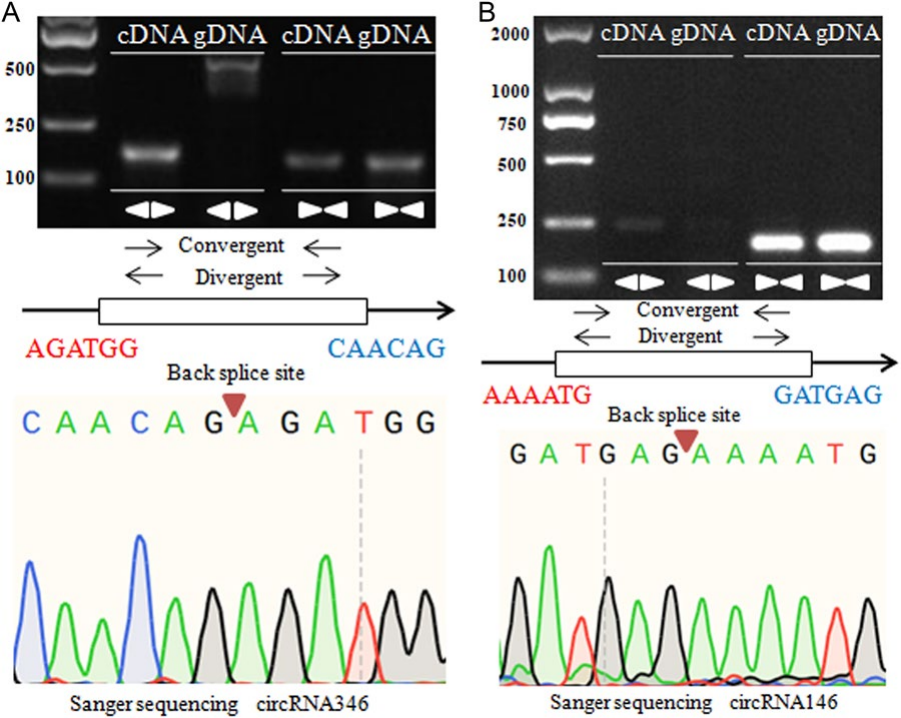
Experimental validation of two cotton circRNAs via PCR amplification and Sanger sequencing. **A** Intergenic circRNA346. **B** Exonic circRNA146

Subsequently, we performed further experiments to test and confirm the circRNA predictions in the cotton pollen grains. The convergent and divergent primers were utilized to amplify the back-splice sites from complementary DNA (cDNA) as well as genomic DNA (gDNA). However, the target PCR bands amplified by divergent primers were collected for further validation via Sanger sequencing. Our results successfully confirmed eight of 12 randomly selected circRNAs (66.7%), including two (50%) exonic circRNAs (circRNA146 and circRNA26) and (75%) intergenic circRNAs, which reveals that our circRNAs predictions contained a relatively high level of accuracy (Figs. 4, S1). For instance, the intergenic circRNA346 and exonic circRNA146 were only amplified by the target PCR products from cDNA with divergent primers but not from genomic DNA. In contrast, these two circRNAs were amplified from both cDNA and genomic DNA with convergent primers (Fig. 4). Additional experimental validated results of the other six cotton circRNAs via PCR amplification and Sanger sequencing were shown in Additional file 2: Fig. S1.

**Fig. 5:**
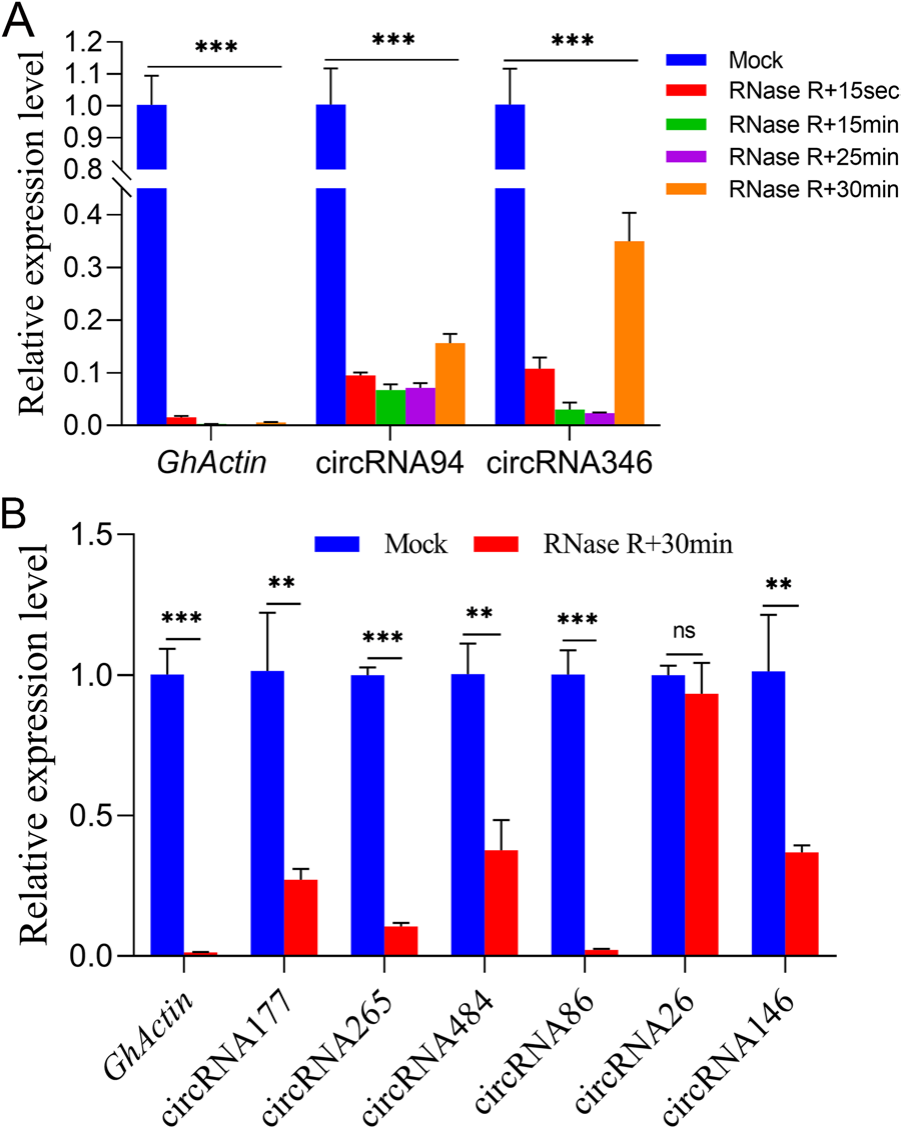
Further qRT-PCR analysis confirmed the relatively stable expression of cotton circRNAs. **A** The qRT-PCR analysis of RNase R (−) (Mock) and RNase R (+) treated total RNA shows that two circRNAs are more RNase R-resistant than linear mRNA in RNase R (+) enzyme treated with four different times. **B** The qRT-PCR analysis shows that the other five circRNAs are more RNase R-resistant than linear mRNA in RNase R (+) with 30 min treatment, except for the circRNA86. Data are presented as the means ± standard deviation (SD) of three biological replicates. Asterisks signify a significant difference as determined by Student’s *t*-test (**P < 0.01; ***P < 0.001; ns, not significant)

To exclude the interference of potential PCR artifacts, the same total RNA samples were further treated with the exonuclease RNase R [RNase R (+)] for 15 sec, 15min, 25min, and 30min to degrade linear RNA molecules as well as without the RNase R-treatment [RNase R (−)] as the Mock. The quantitative real-time polymerase chain reaction (qRT-PCR) analysis of circRNA94 and circRNA346 along with linear mRNA *GhActin* was first performed using the above five treated total RNA samples. The analysis determined a significant decrease in the expression levels of both circRNAs at four different times of RNase R (+) enzyme treatments compared to their corresponding Mocks, while the linear controls were almost completely degraded in all RNase R (+) samples. In addition, these two circRNAs were found to be relatively enriched under RNase R (+) treatment for 30 min compared to other treatments (Fig. 5A). Therefore, the other six circRNAs validated above were treated with RNase R (+) for only 30min before qRT-PCR analysis. Similarly, their results revealed that five circRNAs except circRNA86 were more RNase R-resistant than linear mRNA *GhActin*(Fig. 5B). In brief, these findings predict that circRNAs most probably represent a group of relatively stable transcriptional and posttranscriptional regulators which modulate pollen fertility of cotton CMS-D2 restorer line under HT stress.

### Tissue-specific expression patterns of circRNAs validated by qRT-PCR analysis

**Fig. 6:**
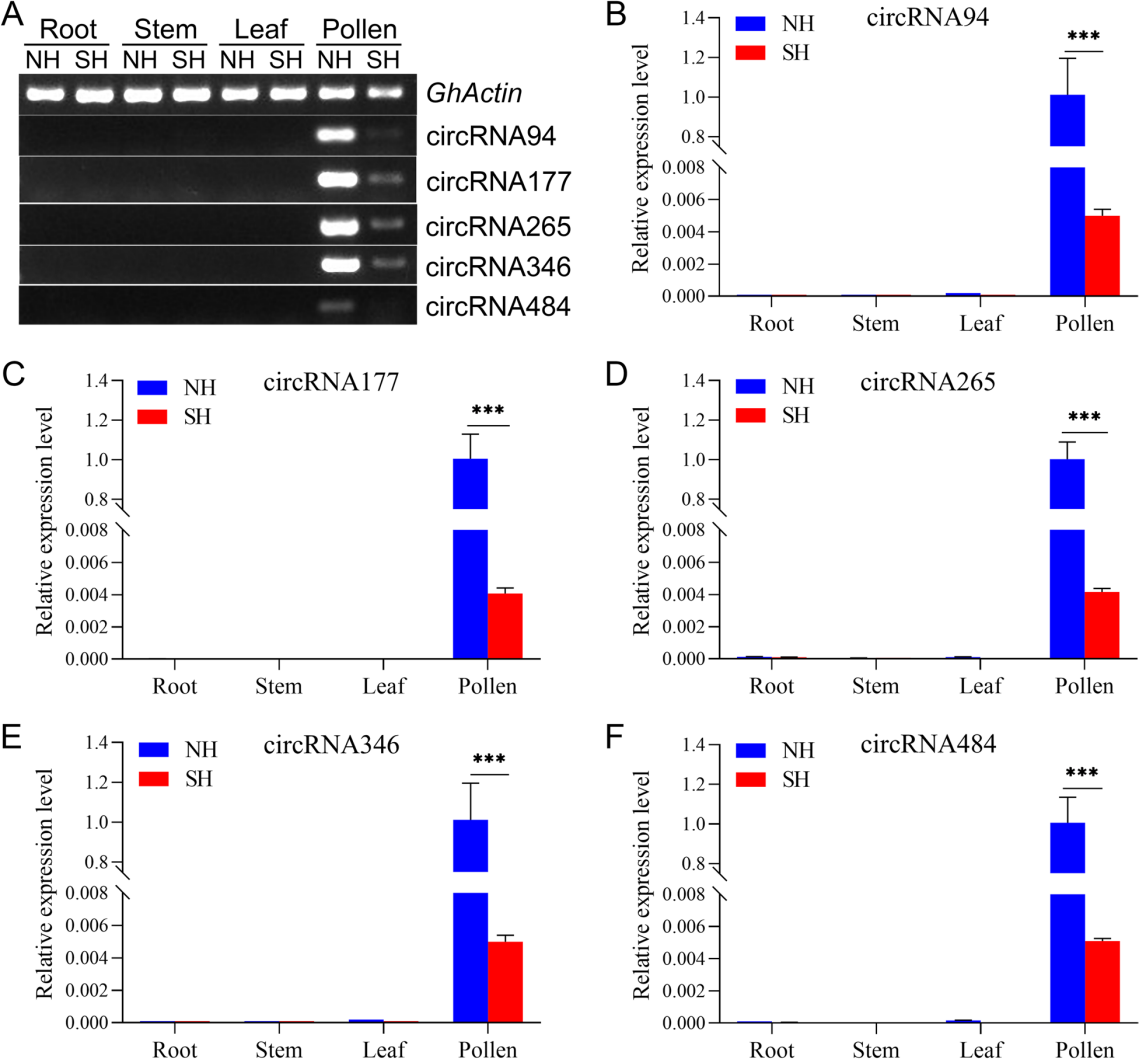
Tissue-specific expression patterns of five circRNAs verified by RT-PCR and qRT-PCR. **A** RT-PCR analysis of five circRNAs in roots, stems, leaves, and pollen of NH and SH. **B**-**F** The qRT-PCR validation of the relative expression levels of five circRNAs in different tissues of NH and SH. *GhActin* is used as a linear control and internal reference gene for normalization. Values are shown as the means ± SD, and the error bars represent the SD of the mean of 2^–ΔΔCt^ with three biological replicates, with NH pollen as a control. Asterisks indicate statistically significant differences between NH and SH (***P < 0.001, Student *t*-test)

Currently, it has become a consensus that circRNAs are tissue preferentially expressed [1, 26, 30]. To confirm this, five circRNAs validated above were first selected for RT-PCR analysis using divergent primers to test the expression levels in roots, stems, leaves at the seedling stage, and pollen grains of NH and SH under HT. As shown in Fig. 6A, all these five circRNAs were only expressed in pollen but had no obvious expression in roots, stems, and leaves of NH and SH. Especially, the circRNA94, circRNA177, circRNA265, and circRNA346 were highly expressed in NH pollen. Further qRT-PCR analysis proved the expression levels of these five circRNAs were significantly higher in the pollen of NH than in SH under HT stress (Fig. 6B-F). This suggests that these circRNAs might participate in the fertile pollen development process of CMS-D2 cotton in response to HT.

### Functional annotation analysis of parental genes of DECs

**Fig. 7:**
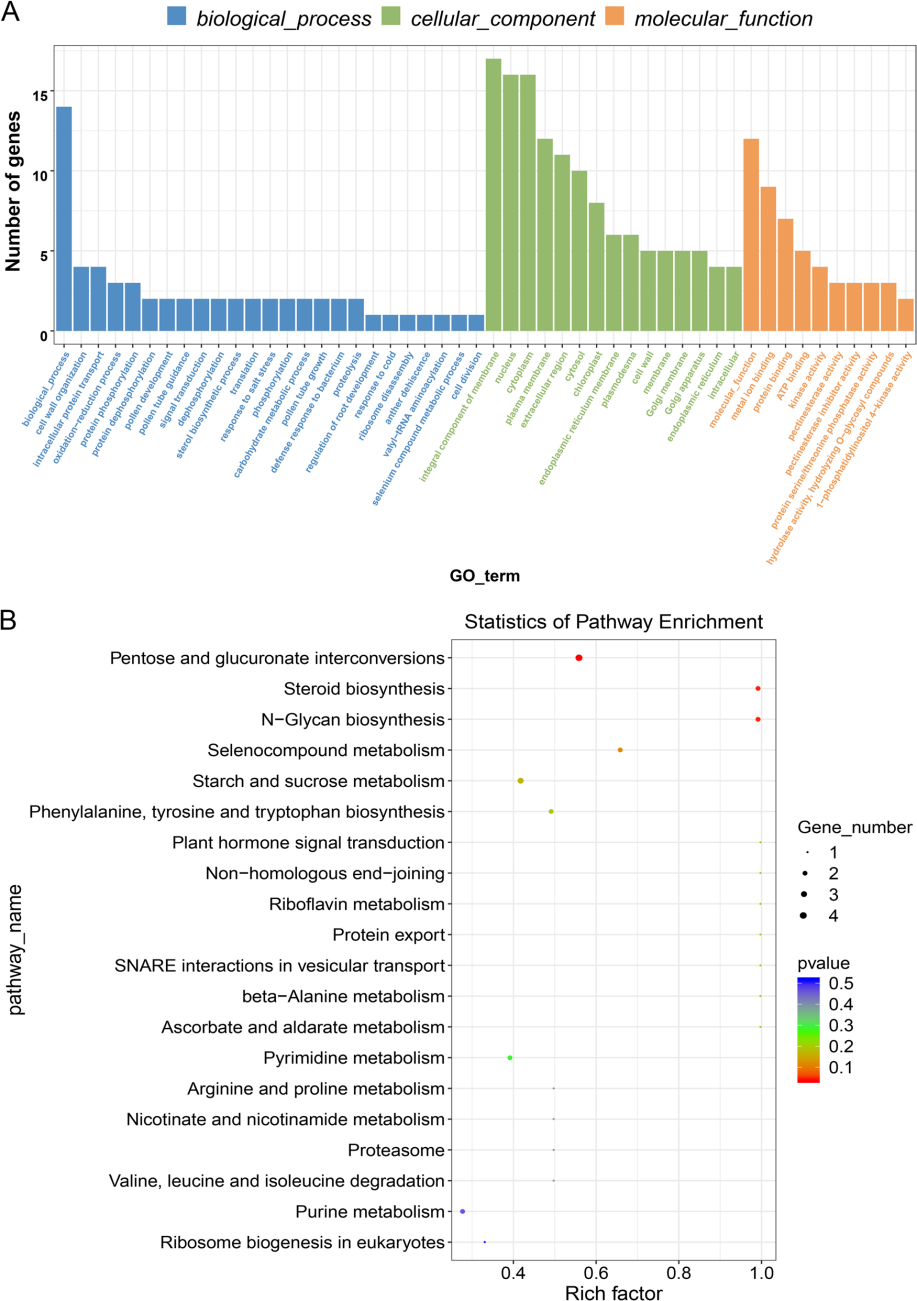
GO functional classification (**A**) and KEGG pathway enrichment (**B**) analysis of parental genes of DECs

To investigate the possible functions of circRNAs and whether the DECs involved in the regulation of pollen fertility restoration stability for CMS-D2 cotton under HT stress, the parental or hosting genes of the DECs were first predicted. A total of 92 parental genes were obtained from the 250 DECs, and GO classification divided these genes into 290 functional GO terms. Thereinto, three main functional classifications of GO categories such as molecular function cellular component, and biological process possessed 110, 50, and 130 functional terms, respectively (Fig. 7A, Additional file 3: Table S2). In molecular functions, the three most significantly enriched categories were hydrolase activity, and hydrolyzing O-glycosyl compounds (GO: 0004553), structural constituent of cell wall (GO: 0005199), and phosphatase activity (GO: 0016791) (*P* ≤ 0.05). Among the cellular components, only two GO terms including extracellular region (GO: 0005576), and endoplasmic reticulum membrane (GO: 0005789) showed significant enrichment. For the biological processes, the four significantly enriched GO terms were pollen tube guidance (GO: 0010183), sterol biosynthetic process (GO: 0016126), dephosphorylation (GO: 0016311), and cell wall organization (GO: 0071555) (Additional file 3: Table S2). Notably, the other predominant GO terms related to pollen and/or anther development were pollen development (GO: 0009555), pollen tube growth (GO: 0009860), pollen tube tip (GO: 0090404), pollen tube (GO: 0090406), anther dehiscence (GO: 0009901), inflorescence development (GO: 0010229), pollen exine formation (GO: 0010584). Besides, induced systemic resistance, jasmonic acid mediated signaling pathway (GO: 0009864), response to ethylene (GO: 0009723), and response to abscisic acid (GO: 0009737) were other functional enriched terms (Additional file 3: Table S2). These functional annotations specify that some circRNAs might contribute to the pollen fertility of cotton CMS-D2 restorer line under HT stress by regulating the parental genes involved in an array of functions related to plant hormone signal transduction.

To further gain insight into the biological functions of DECs, the KEGG pathway enrichment analysis of 39 parental genes was further performed and these genes were annotated with 37 pathways (Additional file 4: Table S3). Among these pathways, four parental genes were assigned to the pentose and glucuronate interconversions pathway and three parental genes showed annotation with the starch and sucrose metabolism pathway. The other pathways only contained one or two parental genes. The three most significantly enriched pathways included pentose and glucuronate interconversions, steroid biosynthesis, and N-Glycan biosynthesis, with a corrected *P*-value < 0.05. Furthermore, the other KEGG pathways related to pollen and/or anther development were also annotated including plant hormone signal transduction and ascorbate and aldarate metabolism (Fig. 7B). These pathways mainly possessed parental genes having biological functions related to sugar and lipid synthesis and metabolism, plant hormone signaling, and ROS scavenging.

Based on functional annotations of parental genes, qRT-PCR technology was utilized to analyze the expression patterns of the corresponding parental genes of the two exonic DECs identified above, named circRNA26 and circRNA146. Thereinto, *Ghir_D10G016080.2*, the parental gene of circRNA26, was a homolog of the vacuole membrane protein KMS1-like (*KMS1*) gene in *A. thaliana,* and *Ghir_D13G010950.1*, the parental gene of circRNA146, was an ortholog of the 5-methyltetrahydropteroyltriglutamate--homocysteine methyltransferase-like (*METE*) gene involved in cysteine and methionine metabolism and seleno compound metabolism. Both *KMS1* and *METE*, as well as the corresponding DECs, were significantly down-regulated in SH than that in NH under HT stress (Additional file 5: Fig. S2). Therefore, the differences in expression levels of circRNAs may influence the functions of pollen fertility restoration for CMS-D2 cotton under HT by interacting with their parental genes.

### Prediction of circRNA‑associated ceRNA networks involved in regulating the pollen fertility under heat stress

**Fig. 8:**
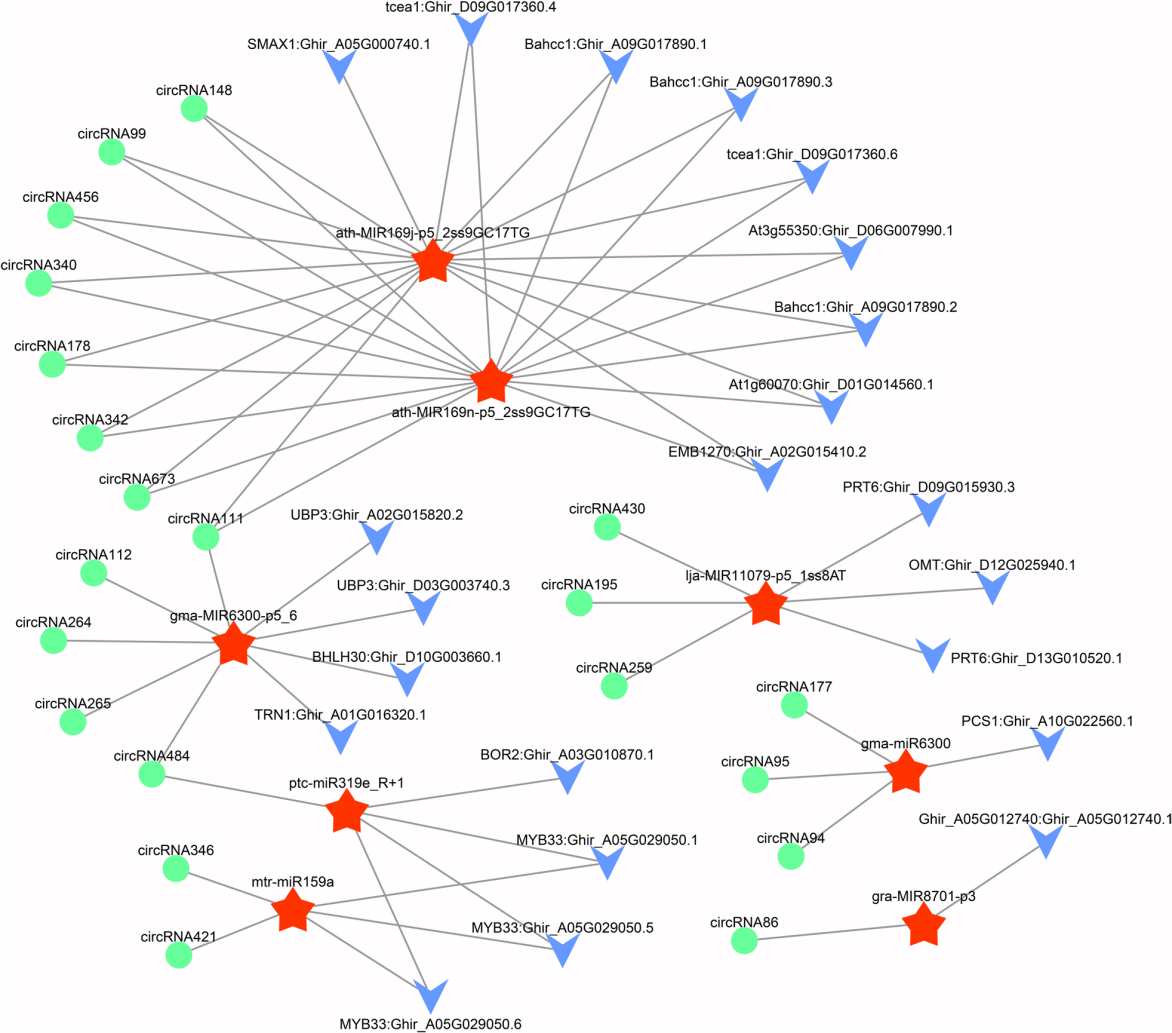
The prediction of circRNA-miRNA-mRNA interaction network involved in the regulation pollen fertility stability of cotton CMS-D2 restorer line under HT stress. The green circle, red pentagram, and blue vee represent the differentially expressed circRNAs and miRNAs; as well as their corresponding mRNA targets, respectively

CircRNAs have been demonstrated to act as miRNA sponges and inhibit miRNA activity by competing with endogenous RNA (ceRNA) networks, thereby regulating gene expression [17]. To confirm if circRNAs have a similar function in cotton CMS-D2 restorer line under HT stress, we first predicted all the potential binding sites of miRNAs for DECs and then identified the candidate ceRNA pairs (circRNA targeting miRNA and miRNA targeting mRNA). Combined with our small RNA sequencing, transcriptome, and degradome data (unpublished), the candidate ceRNA pairs were then screened through DEG analysis, considering that the circRNAs and target genes should have similar expression patterns under HT stress. Finally, a complex circRNA-miRNA-mRNA interaction network for pollen fertility was constructed and delineated by Cytoscape software (Fig. 8). Interestingly, 21 differentially expressed circRNAs contained eight predicted circRNA-binding miRNAs and their corresponding 22 mRNA targets had statistically significant differences among different samples. Among these circRNAs, a considerable number (12, 57.14%) contained miRNA-binding sites for only one miRNA, followed by 38.10% (8) with binding sites for two miRNAs. However, the remaining one circRNA111 contained several binding sites for three miRNAs, including ath-MIR169j-p5_2ss9GC17TG, ath-MIR169n-p5_2ss9GC17TG, and gma-MIR6300-p5_6 (Fig. 8, Additional file 6: Table S4). These results indicate that a single circRNA could target two or more different miRNAs. Meanwhile, a single miRNA could be targeted by a diverse number of circRNAs. For instance, both ath-MIR169j-p5_2ss9GC17TG and ath-MIR169n-p5_2ss9GC17TG were targeted by eight circRNAs.

The *MYB33* is known to act redundantly with MYB65 and has a facultative role during the earlier stages of tapetal development in *Arabidopsis* [53]. It is worth noting that the two circRNAs confirmed above i.e., circRNA346 and circRNA484 may indirectly regulate the transcription changes of gene encoding *MYB33* transcription factor through competitive binding to mtr-miR159a and ptc-miR319e_R+1, respectively (Fig. 8, Additional file 6: Table S4). However, further research is needed on how these two ceRNA network modules (circRNA346-miR159a-*MYB33* and circRNA484-miR319e-*MYB33*) regulate functional pollen development in restorer line of cotton CMS-D2 under heat stress.

## Discussion

### Genome-wide identification and characterization of circRNAs in cotton pollens under heat stress

Since the first excavation of circRNAs in the model plant *Arabidopsis thaliana* [16], their research has recently been progressive in many other crop species, such as rice [8, 21], wheat [22] and maize [23–25]. The landscape of circRNAs in plants differs and generally depends on tissues, varieties, experimental treatment methods, and identification algorithms [20]. In this study, a total of 967 circRNAs were detected in cotton pollens under heat stress via both CIRCExploter2 and CIRI tools (Fig. 1). This was more or less different in comparison with the number of circRNAs identified in previous studies on upland cotton circRNAs, i.e., ovules and leaves: 499 circRNAs [27]; roots and stems: 686 circRNAs; epidermal cells from the ovules with attached fibers at 0 and 5 days post anthesis (DPA): 2,262 circRNAs [54]; ovules (0 DPA), fibers (8 DPA) and stems (seedling stage): 2,811 circRNAs [28]; and also ovules, fibers, and young leaves: 340 high-confidence circRNAs [48]. This divergence is mainly due to differences in identification tools, tissues and varieties utilized to perform analysis. It was observed that pollen grains of CMS-D2 cotton have shown a diverse number of circRNAs, but less overlapped and more specific circRNAs were detected in pollen samples under HT stress (Fig. 1A). This implies that heat stress may affect pollen fertility by directly inducing the expression of more novel circRNAs, rather than only regulating the expression abundance (up- or down-regulation) of existing circRNAs in plant cells [36].

Among the detectable circular RNAs, the proportions of exonic circRNAs were relatively higher than intronic or intergenic circRNAs (Fig. 1B), indicating that circRNAs may be mainly generated from the exonic regions in the cotton genome. This finding was consistent with the previous reports in cotton [27, 28, 48]. Furthermore, there is currently no evidence to support that the distribution of circRNAs is linked with the characteristics of chromosomes. Here, circRNAs in cotton pollens were also found to be almost evenly distributed across the chromosomes between At- and Dt-subgenomes of upland cotton (489 and 465 circRNAs, respectively) (Fig. 1C), indicating the contributions of the two subgenomes to generating the circRNAs may be approximately equal in cotton [47, 48]. Given the lengths of the identified circRNAs in cotton pollen grains, most (75.9%) were shorter than 2,000 bp (Fig. 1D). This phenomenon was also described in the previous studies in cotton [28, 48] and other plant species, e.g., rice [36] and soybean [30].

### Extensively differential expression and tissue specificity of heat-responsive circRNAs in cotton

Recent research in model plant *Arabidopsis* have identified 1,599 previously-unknown and 1,583 heat-specific circRNAs through RNA-sequencing and bioinformatic analysis, and many more circRNAs are induced to express under heat stress than in control condition [38]. Here, a total of 250 DECs were identified in cotton pollens under heat stress, and the proportions of exonic DECs on the At- or Dt-subgenomes showed relatively larger than intronic or intergenic DECs (Fig. 2), indicating that the extensive transcription changes of circRNAs induced by HT may act an important role in regulating the stability of pollen fertility [36]. The genomic mapping exhibited that the 250 DECs were unevenly distributed and most were clustered at the end of chromosomes (Fig. 3), which was consistent with the recent research on cotton [28]. Nevertheless, no DECs were found to be located in the fine-mapped interval of the *Rf*_*1*_ gene as expected [52].

There is currently increasing evidence that the expression of circular RNA has tissue or developmental stage specificity [1, 26, 30, 36]. In this study, it was found that the expression levels of five circRNAs validated above in pollens were all significantly higher than that in roots, stems and leaves of both NH and SH (Figs. 4-6), indicating that these circRNAs have tissue-specific expression patterns, and they appear to be more active in the reproductive growth phase than in the vegetative growth phase of cotton. This finding is in agreement with the previous reports on cotton [27, 48]. Furthermore, these circRNAs showed extremely significantly down-regulated expression in HT-sensitive SH pollens than that in NH under HT stress (Fig. 6), implying that circRNAs may contribute to maintaining the stability of pollen fertility restoration for CMS-D2 cotton in response to heat stress.

### Potential biological roles of the circRNAs involved in pollen fertility stability under heat stress

Previous studies have revealed that some circRNAs participate in regulating the expression of their corresponding parental genes [18, 21, 38]. In plants, it has been reported that expression patterns of some circRNAs present a significant positive correlation with their corresponding parental genes [8, 29, 38]. Consistently, we found two exonic DECs verified in the present study, namely circRNA26 and circRNA146, as well as their corresponding parental genes *KMS1* and *METE*, respectively, were significantly down-regulated in HT-sensitive SH under heat stress (Additional file 5: Fig. S2). However, overexpression of plant circRNAs can also decrease the expression levels of their parental genes [21], indicating that circRNAs may also function as negative regulators of their parental genes [38]. In our study, to further understand the potential roles of circRNAs in CMS-D2 cotton, GO functional classification and KEGG pathway enrichment analysis were also conducted for the parental genes of DECs in NH and SH under heat stress. Based on the assigned GO functional terms, some parental genes were classified into the significantly enriched categories (*P* ≤ 0.05), such as structural constituent of cell wall (GO: 0005199) and phosphatase activity (GO: 0016791) in molecular functions, extracellular region (GO: 0005576) and endoplasmic reticulum membrane (GO: 0005789) in cellular components, as well as pollen tube guidance (GO: 0010183), sterol biosynthetic process (GO: 0016126), dephosphorylation (GO: 0016311) and cell wall organization (GO: 0071555) in biological processes (Fig. 7A, Additional file 3: Table S2), implying that circRNAs may contribute distinctly during anther and/or pollen development via associating with different cellular biological processes [36]. Remarkably, the other GO terms linked with pollen and/or anther development, including pollen development (GO:0009555) , pollen tube growth (GO: 0009860), pollen tube tip (GO: 0090404) , pollen tube (GO: 0090406), anther dehiscence (GO: 0009901), inflorescence development (GO: 0010229), pollen exine formation (GO: 0010584) , were also annotated, suggesting the involvement of circRNAs in modulating the pollen fertility stability under HT stress. Besides, induced systemic resistance, and jasmonic acid mediated signaling pathway (GO: 0009864), response to ethylene (GO: 0009723), and response to abscisic acid (GO: 0009737) were also enriched during GO analysis (Additional file 3: Table S2). It has been reported that the main endogenous phytohormones including jasmonic acid (JA) [55], auxin [56, 57], and gibberellin acid (GA) [58] participate in the response to HT during anther development. Therefore, we infer that some circRNAs may play essential roles in maintaining the pollen fertility stability of cotton CMS-D2 restorer line under heat stress via regulating the parental genes involved in plant hormone signal transduction [50]. KEGG pathway analysis of parental genes of DECs showed that they were significantly enriched in Pentose and glucuronate interconversions, Steroid biosynthesis, and N-Glycan biosynthesis pathways (Fig. 7B, Additional file 4: Table S3). Consistently, a recent study in soybean has also found that the Pentose and glucuronate interconversions pathway is related to pollen development under HT stress [59]. Moreover, the other KEGG pathways associated with pollen and/or anther development were also enriched, including Plant hormone signal transduction and Ascorbate and aldarate metabolism (Fig. 7B). Taken together, these pathways predominantly belong to sugar and lipid synthesis and metabolism, plant hormone signaling, and ROS scavenging, indicating that circRNAs may play critical roles in the fertility restoration stability for CMS-D2 in cotton under heat stress through integrated sugar and lipid, plant hormone, and ROS signals.

Recent evidence in human and animals has demonstrated that circRNAs may act as probable miRNA sponges, which can sequester miRNA away from its corresponding mRNA targets by circRNA-miRNA-mRNA networks, thus regulating gene expression [17]. For instance, an abundant *circHIPK3* could regulate cell growth by acting as sponges for nine miRNAs with 18 potential binding sites in human cells [60].In plants, although many circRNAs acting as miRNA sponges have been magnificently predicted, almost no direct experimental evidence has been provided so far [28, 30, 36–38, 48]. In the present study, we have also constructed a putative circRNA-mediated ceRNA network involved in regulating pollen fertility stability of cotton CMS-D2 restorer line under heat stress, encompassing 21 DECs, eight predicted circRNA-binding miRNAs as well as their corresponding 22 mRNA targets (Fig. 8, Additional file 6: Table S4). Notably, a single circRNA could target two or more different miRNAs, and meanwhile, a single miRNA could be also targeted by diverse circRNAs. This finding was consistent with previous studies in cotton [28, 48] and other plant species, e.g., *Arabidopsis* [38] and rice [36]. MiR159 and miR319 are well-studied highly conserved miRNAs that participate in vegetative development, reproduction, and hormone regulation in plants [61]. In *B. campestris*, it has been reported that overexpressed MIR159a and MIR319c can contribute to late anther development and promote pollen abortion [62]. Previous study have shown that *AtMYB33* and *AtMYB65* genes in *Arabidopsis* act a redundant role in regulating anther development, and the tapetum cells of *atmyb33* and *atmyb65* double mutants produce excessive vacuolization, swelling, and hypertrophy before meiosis, ultimately leading to pollen abortion [53]. A recent study in cotton has also confirmed that the miR319c-*MYB33* module is involved in regulating the trade-off between plant growth and defense in *Verticillium dahliae* infection [63]. Considering the roles of the two circRNAs confirmed in this study, i.e., circRNA346 and circRNA484 may indirectly regulate the expression of *MYB33* target by competitive binding to mtr-miR159a and ptc-miR319e_R+1, respectively (Fig. 8, Additional file 6: Table S4). Hence, we imply that circRNA346 and circRNA484 are involved in the regulation of pollen fertility stability under heat stress. Although many circRNAs have been identified in plants by the large-scale sequencing, only several circRNAs have been clarified with clear biological functions so far [20]. In rice, at least two independent T_1_ deletion lines for the EIciRNA *Os06circ02797* and the intergenic circRNA *Os05circ02465* were produced with a multiplexed CRISPR-Cas9 strategy, and their expression levels of parental or flanking genes were not influenced. Two deletion lines of *Os05circ02465* exhibited high salt tolerance with significantly lower germination rates. Seedlings of the *Os06circ02797* mutants *Os06circ02797D1* and *Os06circ02797D2* presented a rapid growth phenotype with higher chlorophyll *a*/*b* contents after seed germination [64]. Therefore, in-depth validation experiments should be implemented on circRNA346 and circRNA484 using a multiplexed CRISPR-Cas9 strategy, aiming to further reveal the underlying molecular mechanisms of how these two key ceRNA network modules (circRNA346-miR159a-*MYB33* and circRNA484-miR319e-*MYB33*) partake in modulating the stability of pollen fertility restoration for CMS-D2 in cotton under heat stress.

## Conclusions

To our knowledge, this study systematically investigated the abundance and characteristics, expression patterns, and potential functions of circRNAs during cotton pollen development in response to HT stress for the first time by high-throughput deep sequencing technology. Functional annotation of the parental genes of DECs indicated that circRNAs and their mediated ceRNA networks might play important regulatory roles in pollen fertility stability of the CMS-D2 restorer line under heat stress via interlinking with the sugar and lipid, plant hormone and ROS signals. In short, our results revealed that circRNAs may be one of essential regulators in the stability of pollen fertility restoration for CMS-D2 in cotton under HT stress, which will shed new light on further clarifying the complex regulatory mechanisms of the negative effects of sterile cytoplasm on the pollen/anther development of CMS-D2 restorer line or "three-line" hybrid cotton.

## Methods

### Plant materials and sample collection

In this study, one set of isonuclear alloplasmic near-isogenic cotton restorer lines NH [N(*Rf*_*1*_*rf*_*1*_)] and SH [S(*Rf*_*1*_*rf*_*1*_)] were chosen as materials, whose pollen fertility stability presented obvious differences in performance under continuous heat stress in the field [50]. Specifically, NH with the normal *G. hirsutum* cytoplasm was HT-tolerant, whereas the anther and/or pollen development of SH (formerly also named ZBR) carrying the sterile *G. harknessii* (CMS-D2) cytoplasm was highly sensitive to HT stress [46, 50]. Thus, NH and SH are near-isogenic lines (NILs) of isonuclear alloplasmic type with a similar nucleus but different cytoplasm. All cotton materials were developed in detail as described in our previous studies [49, 52, 65], and the seeds were obtained from the Cotton Heterosis Utilization Laboratory, National Key Laboratory of Cotton Bio-breeding and Integrated Utilization, Institute of Cotton Research of Chinese Academy of Agricultural Sciences (ICR-CAAS), Anyang, China.

During the summer of 2020, NH and SH were grown at the Baibi East Experimental Farm, ICR-CAAS, Anyang, Henan Province (36°10′N, 114°35′E), and the experimental field of the Cotton Research Institute of Jiang Xi Province, Jiujiang, Jiangxi Province (29°71′N, 115°85′E) are located in two main cotton-producing areas of the Yellow River basin and the Yangtze River basin in China, respectively. All cotton field management practices were according to local recommendations. In late July and early August, mature pollen grains were harvested in three biological replicates per line from Anyang (AP) and Jiujiang (JP) under continuous mild and extreme HT stress, respectively. Thereinto, the specific duration of HT stress and standardized sample naming have been described in detail in our recent study [50]. Meanwhile, the roots, stems and leaves of NH and SH were also collected at the seedling stage. All the tissues were immediately frozen in liquid nitrogen and then stored at −80°C in a freezer for further use.

### Total RNA extraction and ribonuclease R (RNase R) treatment

Total RNA was isolated from each pollen sample using the TIANGEN RNAprep Pure Plant Plus Kit (Polysaccharides & Polyphenolics-rich; DP441) according to the manufacturer's procedure. The DNase I present in the kit was utilized to remove DNA contamination. The quantity and quality of RNA were assessed with NanoDrop ND-1000 (NanoDrop, Wilmington, DE, USA). The RNA integrity was quantified by an Agilent 2100 Bioanalyzer system (Agilent Technologies, CA, USA) with a RIN number > 7.0.

To prepare RNase R-treated total RNA samples for SH pollens, the purified DNase I-treated total RNA was incubated for 15 sec, 15 min, 25 min, and 30 min, respectively, at 37°C with 3 units per microgram (μg) of RNase R enzyme (Beyotime, Shanghai, China) and then total RNA was heated at 70℃ for 10 min to inactivate RNase R enzyme.

### RNA library construction and sequencing

After initial quality confirmations, about 5 μg of total RNA was depleted of ribosomal RNA following the manuscript of the Ribo-Zero™ rRNA Removal Kit (Illumina, San Diego, USA). The remaining RNAs were then fragmented into small pieces with divalent cations under HT. Subsequently, the cleaved RNA fragments were reverse-transcribed to yield the purified cDNA libraries with standard quality. The quality of final cDNA libraries was again checked by the Agilent 2100 Bioanalyzer system (Agilent Technologies, CA, USA), and the average insert size for the final libraries was 300 bp (± 50 bp). The libraries after cluster generation were sequenced on an Illumina Novaseq™ 6000 platform (LC Bio, Hangzhou, China) according to the vendor's recommended protocol, and finally, 150 bp paired-end sequencing reads were generated for downstream analysis.

### Identification of candidate circRNAs

After RNA sequencing, Cutadapt software [66] was first used to filter the reads that contained adaptor contamination, undetermined bases, and low-quality bases from the raw data (raw reads in fastq format). Subsequently, the sequence quality (Q20, Q30) and GC content of the clean data for each sample were calculated with FastQC (http://www.bioinformatics.babraham.ac.uk/projects/fastqc/). Here, we used both Bowtie2 [67] and Hisat2 [68] to map clean reads to the cotton reference genome of TM-1 [69]. Further, the Tophat-fusion was applied to map the unmapped reads to the cotton reference genome [70]. Afterward, both CIRCExplorer2 [3, 13] and CIRI [71] software were used to *denovo* assemble the mapped reads to circular RNAs at first, and then back-splicing reads were also identified in unmapped reads by Tophat-fusion [70]. The threshold criteria for circRNA identification were adopted as follows: (1) mismatch bases ≤ 2; (2) back-spliced junction reads ≥ 1; (3) the distance between two splice sites on the genome is less than 100 kb. Finally, all unique circRNA candidates were generated for each sample according to the above screening criteria.

### Differential expression analysis of circRNAs

The expression levels of circRNAs in each sample were first calculated based on the counts of junction read and then normalized with the following transcript per million (TPM) criteria [72]: Normalized expression level = (mapped reads) / (total reads) * 1,000,000. The differentially expressed circRNAs (DECs) among desired pollen grains samples were identified with the cutoff threshold of |log2 (fold change)| ≥ 1 and statistical significance (adjusted *P*-value < 0.05) in edgeR [73].

### Functional annotation of the parental genes of DECs

The potential functions of the parental genes of DECs were retrieved from Gene Ontology (GO) and Kyoto Encyclopedia of Genes and Genomes (KEGG) enrichment databases. The GOseq R package [74] was used for GO functional categories analysis, and the KOBAS software [75] was used to test the statistical enrichment of parental genes of the DECs in the KEGG pathways. The functional annotations with a corrected *P*-value < 0.05 were considered statistically significant.

### CircRNA-associated competing endogenous RNA (ceRNA) network prediction

Independently developed scripts in-house and Ssearch36 software (36.3.6) was used for circRNA-miRNA interaction analysis by following the rules of targetmics (LC Bio, Hangzhou, China). For the miRNA-mRNA interaction pairs, the mRNA genes targeted by the most abundant miRNAs were predicted by PsRobot software [76], and computational target prediction algorithms TargetFinder [77] were used to identify miRNA binding sites, with the screening criteria of Alignment score value ≤ 4. Lastly, if the circRNA and mRNA shared the same one or more miRNAs, we defined the circRNA as a candidate miRNA sponge for the mRNA gene and hence representative of candidate ceRNA pairs. Meanwhile, the complex circRNA-miRNA-mRNA interaction network was constructed and visualized by Cytoscape software [78] to display the potential interaction connections between those circRNA, miRNA, and mRNA having statistically significant differences among different samples.

### PCR amplification and Sanger sequencing

The circRNA candidates in mature pollen were confirmed using the following procedure. A total of 1 μg total and RNase R-treated RNA of each sample was first used to reverse-transcribe for cDNA synthesis with random primers using a PrimeScript™ RT Master Mix for Perfect Real Time (RR036A, Takara, Japan). Second, the gDNA was isolated from NH cotton using the modified cetyltrimethylammonium bromide (CTAB) method [79], and the gDNA was used as a negative control for divergent primers. Third, a set of the divergent and convergent primers for circRNA candidates identified in this study were designed as described in methods of previous studies [20, 30, 36], while convergent primers were here used as a positive control. All primers were designed and synthesized commercially (BioSune Biotechnology, Shanghai, China), and are listed in Additional file 7: Table S5. Then, both PCR and reverse transcription (RT)-PCR reactions were prepared using 2×Es Taq MasterMix (Dye; CW0690M, Cowin Biotech, Beijing, China) following the manufacturer’s instructions to amplify back-spliced junction sites of objective circRNAs. The specific reaction procedure for PCR or RT-PCR was pre-denaturation at 94 ℃ for 2 min, followed by 32 cycles of denaturation at 94℃ for 30 sec, annealing at 56℃ for 30 sec, and extension at 72 ℃ for 20 sec; and then final extension at 72 ℃ for another 2 min. The target PCR products were separated from 1% agarose gel with the GelRed ^TM^ Nucleic Acid Gel Stain (10,000× in water; 41003, Biotium, USA), and each band was excised and purified with TaKaRa MiniBEST Agarose Gel DNA Extraction Kit Ver.4.0 (9762, Takara, Japan). Finally, Sanger sequencing was performed to further confirm the junction reads of target circRNAs.

### qRT-PCR analysis

Subsequently, the qRT-PCR assays were performed on a Mastercycler ep realplex machine (Eppendorf, Hamburg, Germany) using TransStart^®^ Top Green qPCR SuperMix (AQ131, TransGen Biotech, Beijing, China). The qRT-PCR aliquot contained 1 μL cDNA, 0.4 μL of each forward and reverse primer (10 μM), 10 μL 2×TransStart^®^ Top Green qPCR SuperMix, and 8.2 μL RNase-free ddH2O and the reaction conditions were performed with an initial denaturation at 94 °C for 30 sec, followed by 40 cycles of denaturation at 94 °C for 5 sec, annealing at 55 °C for 15 sec, and extension at 72°C for 10 sec. Additionally, a melting curve was also produced for each sample at the end of each run to determine whether had the specificity of the amplified PCR product. The housekeeping gene *G. hirsutum Actin* (*GhActin*) was used as an internal control for data normalization, and the relative expression levels of circRNAs were calculated with the 2^−ΔΔCt^ method [80], as described in detail by our previous study [65]. To ensure the reliability of the assay data, all the results were obtained from three biological replicates and two technical repetitions. The circRNA-specific divergent primers for qRT-PCR were designed and synthesized commercially (BioSune Biotechnology, Shanghai, China), and are listed in Additional file 7: Table S5.

### Statistical analysis and graphical presentation

Each graphical plot in the present study denotes the results of multiple independent experiments (n ≥ 3), and the values are presented as means ± standard deviation (SD). The statistical significance analyses of circRNA expression between NH and SH under HT were estimated using a two-tailed Student’s *t*-test, and a *P*-value < 0.05 was considered for significant difference. UpSet Venn and Undirected Network diagrams in this manuscript were produced using OmicShare online tools (https://www.omicshare.com/tools/home/soft/getsoft.html). Moreover, an integrative toolkit TBtools [51] was used to graphically present bending heat map.

### Supplementary Information

**Additional file 1: Table S1.** Overview of circRNA sequencing data.

**Additional file 2: Fig. S1.** Experimental validation of the other six cotton circRNAs via PCR amplification and Sanger sequencing. (A) Intergenic circRNA94. (B) Intergenic circRNA177. (C) Intergenic circRNA265. (D) Intergenic circRNA484. (E) Intergenic circRNA86. (F) Exonic circRNA26.

**Additional file 3: Table S2.** GO functional classification of parental genes of differentially expressed circRNAs (DECs).(XLSX 30 KB)

**Additional file 4: Table S3.** KEGG pathway enrichment analysis of parental genes of DECs.

**Additional file 5: Fig. S2.** The qRT-PCR analysis of the relative expression levels of two exonic DECs and their corresponding parental genes in pollen of NH and SH under HT. (A) The relative expression levels of circRNA26 and its parental gene *KMS1*. (B) The relative expression levels of circRNA146 and its parental gene *METE*. *GhActin* is used as an internal reference gene for normalization. Values are shown as the means ± SD, and the error bars represent the SD of the mean of 2^–ΔΔCt^ with three biological replicates, with NH pollen as a control. Asterisks indicate statistically significant differences between NH and SH (**P < 0.01; ***P < 0.001, Student *t*-test).

**Additional file 6: Table S4.** Predicted circRNA-miRNA-mRNA connections for DECs in pollen of NH and SH under HT stress.

**Additional file 7: Table S5.** Divergent and convergent primers used for the validation of eight differentially expressed circRNAs in this study.

**Additional file 8: **Original, unprocessed full-length gel and blot images used in this study.

## Data Availability

Data supporting this study are included within the article and/or supporting materials. The sequencing data have been deposited in the Sequence Read Archive (SRA) at the National Center for Biotechnology Information (NCBI) under the accession number PRJNA1002563.

## Abbreviations

cDNA: Complementary DNA
ceRNA: Competing endogenous RNA
circRNAs: Circular RNAs
CMS: Cytoplasmic male sterility
CMS-D2: CMS conditioned by *Gossypium harknessii* cytoplasm
DECs: Differentially expressed circRNAs
DPA: Days post anthesis
gDNA: Genomic DNA
GO: Gene Ontology
HT: High-temperature
KEGG: Kyoto Encyclopedia of Genes and Genomes
NILs: Near-isogenic lines
PCR: Polymerase chain reaction
qRT-PCR: Quantitative real-time polymerase chain reaction
RNase R: Ribonuclease R
RNase R (+): Treated with the exonuclease RNase R
RNase R (−): Without the RNase R-treatment
ROS: Reactive oxygen species
RT-PCR: Reverse transcription (RT)-PCR
TPM: Transcript per million
