# Supplementary material for: Identification and characterization of circular RNAs involved in the fertility stability of cotton CMS-D2 restorer line under heat stress
Source: BMC Plant Biol. 2024 Jan 5;24:32. doi: 10.1186/s12870-023-04706-w (PMC10768462; doi:10.1186/s12870-023-04706-w)
Supplement: Supplementary file 2 — Additional file 2: Fig. S1. Experimental validation of the other six cotton circRNAs via PCR amplification and Sanger sequencing. (A) Intergenic circRNA94. (B) Intergenic circRNA177. (C) Intergenic circRNA265. (D) Intergenic circRNA484. (E) Intergenic circRNA86. (F) Exonic circRNA26. [file 12870_2023_4706_MOESM2_ESM.docx]

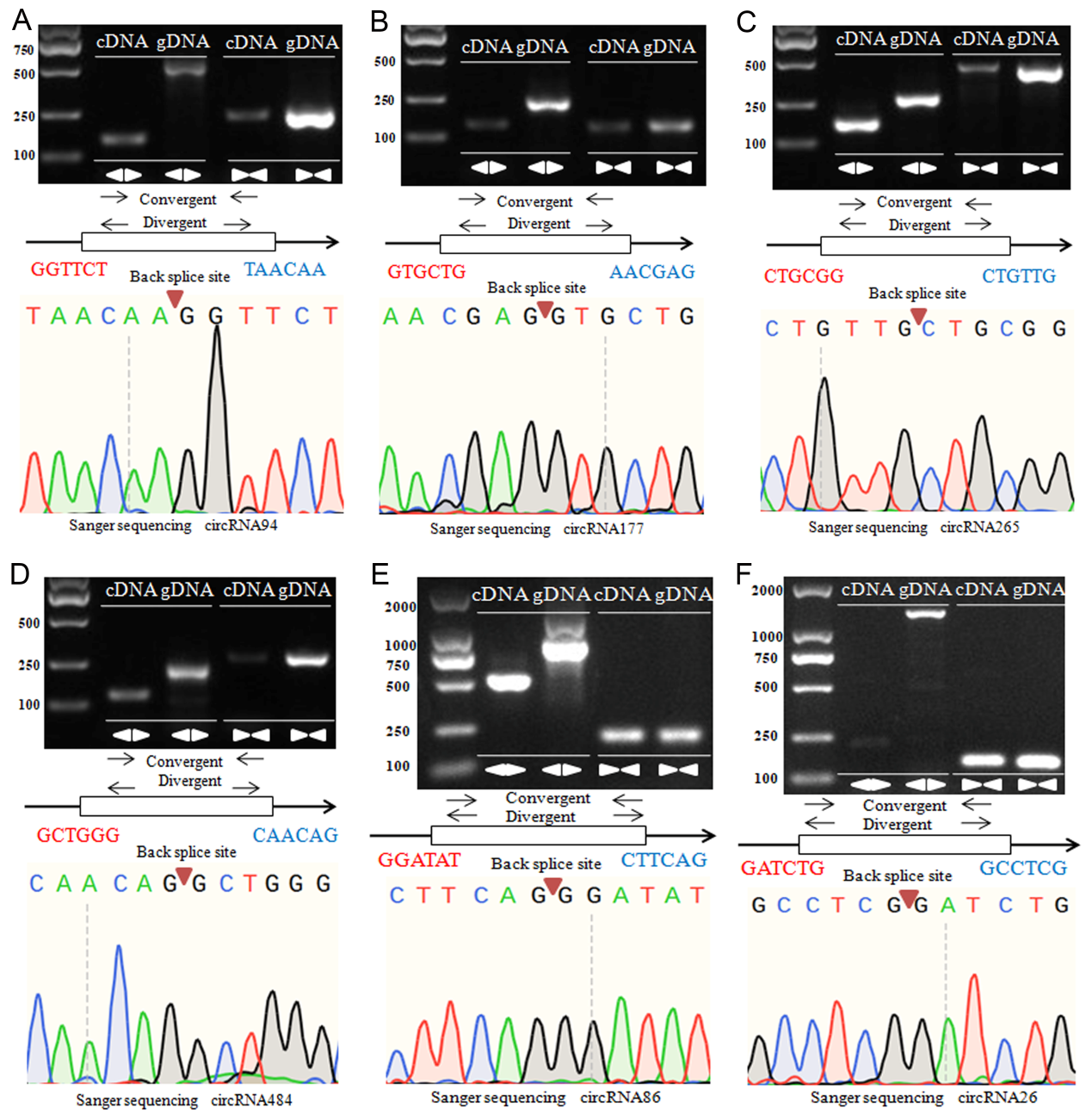


**Additional file 2: Fig. S1.** Experimental validation of the other six cotton circRNAs via PCR amplification and Sanger sequencing. (A) Intergenic circRNA94. (B) Intergenic circRNA177. (C) Intergenic circRNA265. (D) Intergenic circRNA484. (E) Intergenic circRNA86. (F) Exonic circRNA26.
