# Supplementary material for: Identification and characterization of circular RNAs involved in the fertility stability of cotton CMS-D2 restorer line under heat stress
Source: BMC Plant Biol. 2024 Jan 5;24:32. doi: 10.1186/s12870-023-04706-w (PMC10768462; doi:10.1186/s12870-023-04706-w)
Supplement: Supplementary file 5 — Additional file 5: Fig. S2. The qRT-PCR analysis of the relative expression levels of two exonic DECs and their corresponding parental genes in pollen of NH and SH under HT. (A) The relative expression levels of circRNA26 and its parental gene KMS1. (B) The relative expression levels of circRNA146 and its parental gene METE. GhActin is used as an internal reference gene for normalization. Values are shown as the means ± SD, and the error bars represent the SD of the mean of 2–ΔΔCt with three biological replicates, with NH pollen as a control. Asterisks indicate statistically significant differences between NH and SH (**P < 0.01; ***P < 0.001, Student t-test). [file 12870_2023_4706_MOESM5_ESM.docx]

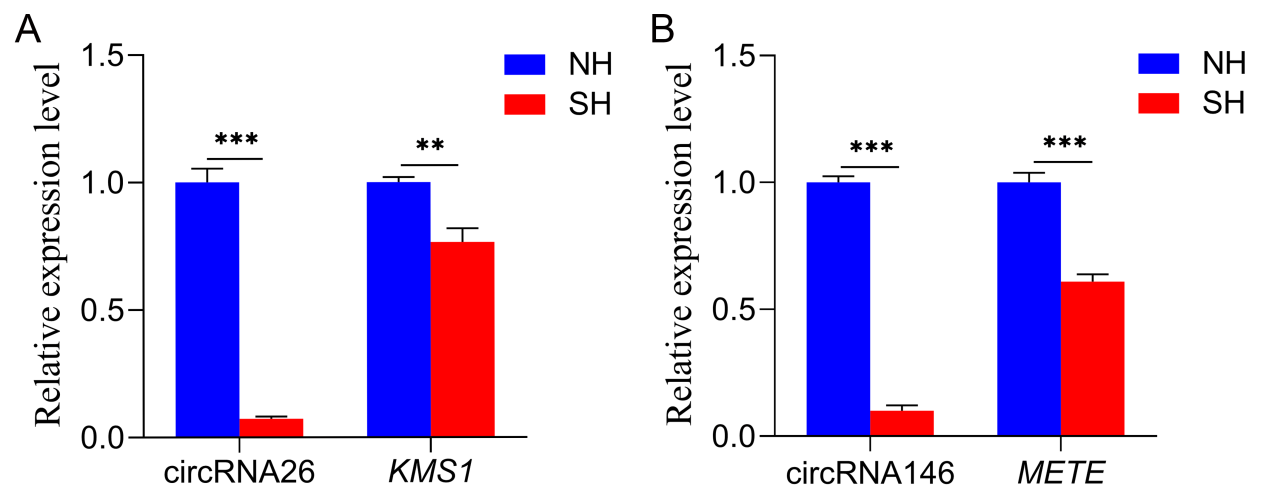


**Additional file 5: Fig. S2.** The qRT-PCR analysis of the relative expression levels of two exonic DECs and their corresponding parental genes in pollen of NH and SH under HT. (A) The relative expression levels of circRNA26 and its parental gene *KMS1*. (B) The relative expression levels of circRNA146 and its parental gene *METE*. *GhActin* is used as an internal reference gene for normalization. Values are shown as the means ± SD, and the error bars represent the SD of the mean of 2^–ΔΔCt^ with three biological replicates, with NH pollen as a control. Asterisks indicate statistically significant differences between NH and SH (***P* < 0.01; ****P* < 0.001, Student *t*-test).
