# Supplementary material for: Identification and characterization of circular RNAs involved in the fertility stability of cotton CMS-D2 restorer line under heat stress
Source: BMC Plant Biol. 2024 Jan 5;24:32. doi: 10.1186/s12870-023-04706-w (PMC10768462; doi:10.1186/s12870-023-04706-w)
Supplement: Supplementary file 8 — Additional file 8: Original, unprocessed full-length gel and blot images used in this study. [file 12870_2023_4706_MOESM8_ESM.docx]

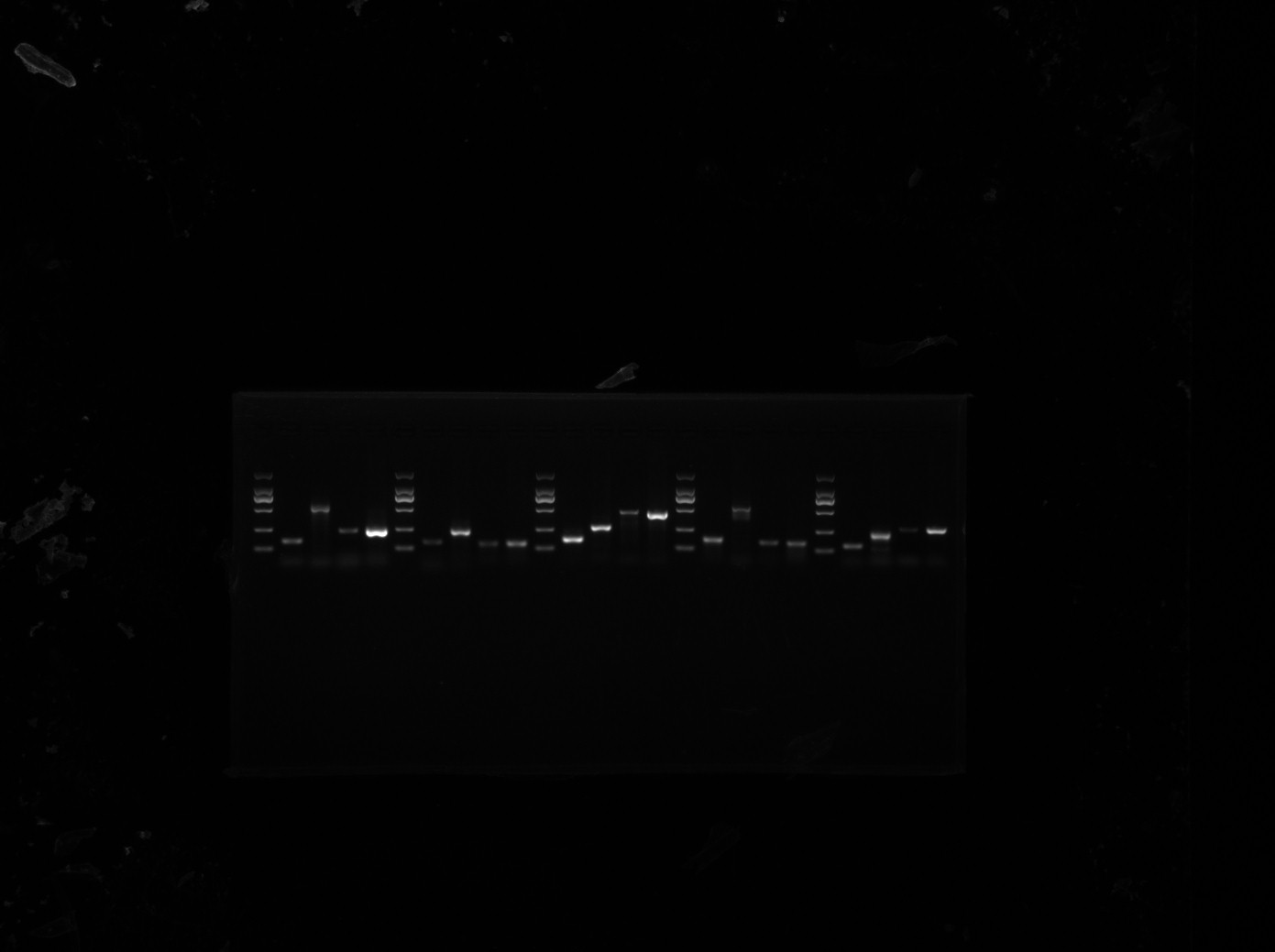


**Figs.4 and S1** From left to right: circRNA94, circRNA177, circRNA265, circRNA346 and circRNA484.


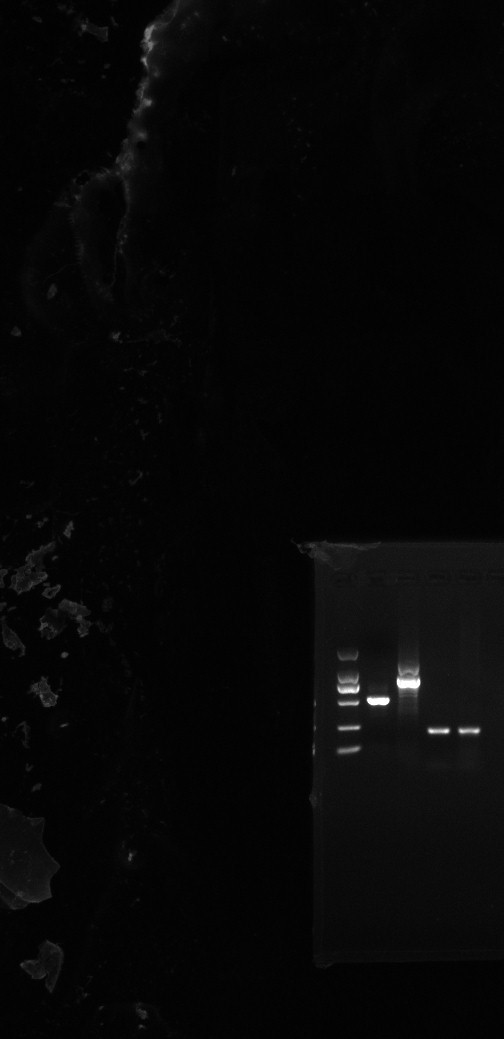

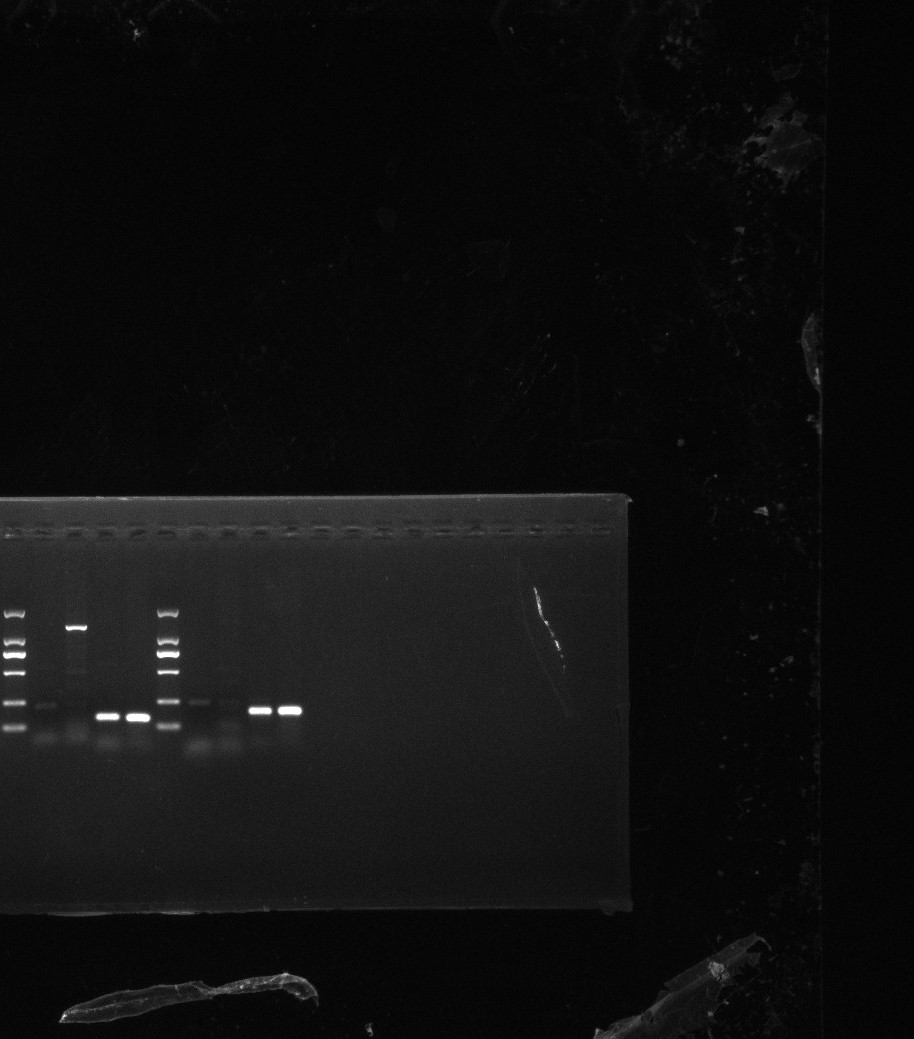


**Figs.4 and S1** From left to right: circRNA86, circRNA26 and CircRNA146.


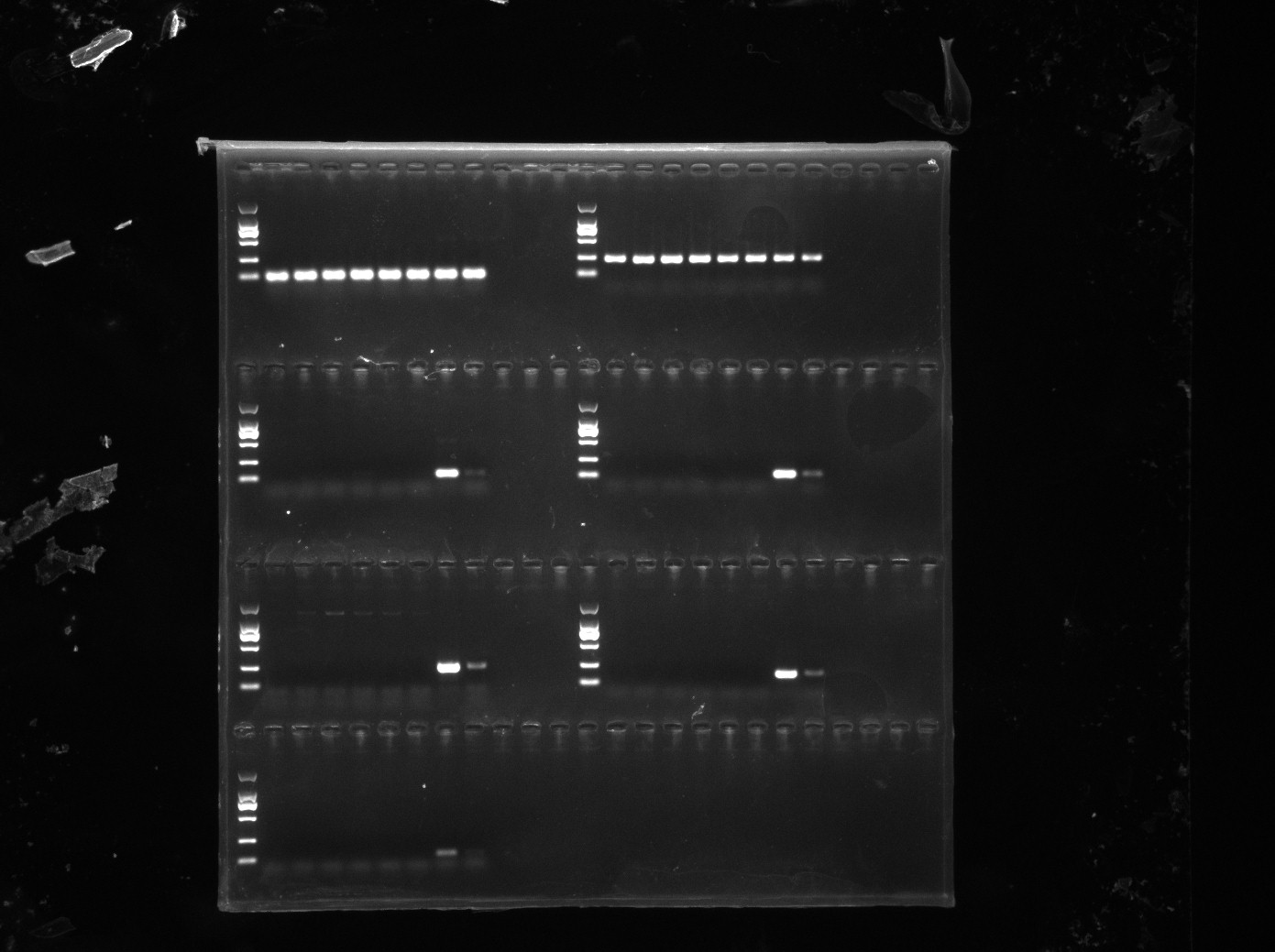


**Fig. 6A** From left to right: *GhU6* (Not presented in this study), *GhActin*, circRNA94, circRNA177, circRNA265, circRNA346 and circRNA484.
